# Use of terlipressin in critically ill children with liver disease

**DOI:** 10.1186/s12882-020-01914-6

**Published:** 2020-08-20

**Authors:** Romit Saxena, Aravind Anand, Akash Deep

**Affiliations:** Paediatric Intensive Care Unit, King’s College Hospital NHS Foundation Trust, Denmark Hill, London, SE5 9RS UK

**Keywords:** Hepatorenal syndrome, Pediatrics, Liver disease, Acute kidney injury, Terlipressin

## Abstract

**Background:**

Terlipressin, a long-acting synthetic analogue of vasopressin has been used in the adult population for various indications including hepatorenal syndrome (HRS-AKI), esophageal variceal hemorrhage (EVH) and shock, but its use in pediatrics is still limited to individualized cases and data on safety and efficacy is scant.

**Methods:**

We reviewed the patient records of children with liver disease and Acute Kidney Injury requiring terlipressin admitted to the Paediatric Intensive Care Unit (PICU) of King’s College Hospital, London from January 2010–December 2017, with special emphasis on its effect on renal parameters and adverse event profile.

**Results:**

Twenty-one terlipressin administration records in a total of 16 patients (median) (IQR) 10 years (6.1–14.4) were included. The drug was initially given as a bolus dose in all cases, followed by either bolus or infusion with median dosage being 5.2 (3.8–6.7) mcg/kg/hour. After administration, a sustained increase of mean arterial pressure was observed. There was an improvement in serum creatinine (Cr) (at 24 h; *p* = 0.386) and increase in urine output (UO), especially in the hepatorenal syndrome subgroup (HRS-AKI). We found minimal evidence of gastrointestinal side effects including feeding intolerance and vasoconstrictive side effects including cyanosis / ischaemia of extremities.

**Conclusion:**

Terlipressin was found to be safe in critically sick children with liver disease with positive impact on renal parameters which might be taken as a surrogate marker of HRS reversal, though effects on outcomes are difficult to ascertain. It is important to be aware of all its side-effects and actively watch for them. Future prospective studies are warranted to validate these findings.

## Background

Terlipressin (triglycyl lysine vasopressin), is a long-acting synthetic analogue of vasopressin. The distribution half-life of terlipressin is 8 min and it gets eliminated in 50 min [1, 2]. Vasopressin and its analogues have been used for indications such as septic shock (especially catecholamine refractory septic shock) [3–6], bleeding esophageal varices [7–9] and hepatorenal syndrome (HRS-AKI) [10, 11]. But, its use and research has been limited predominantly to the adult population. Unique pathophysiology of HRS (splanchnic vasodilatation) justifies the use of a splanchnic vasoconstrictor like terlipressin.

Use of terlipressin in children has been limited to vasodilatory shock [12, 13]. Experience in pediatric population, for indications as hepatorenal syndrome, has been limited to small case series [14]. We retrospectively analyzed case records of children with liver disease treated with terlipressin in our tertiary PICU (Paediatric intensive care unit) in London. The aim of this retrospective study was to analyze changes in hemodynamic, renal parameters and adverse event profile after administration of terlipressin in children with liver disease.

## Methods

A retrospective chart review was conducted using pre-recorded, patient-centered data to study the impact of terlipressin on renal parameters and adverse event profile. We reviewed the patient records of children, admitted to PICU, King’s College Hospital, London who required terlipressin between 2010 and 2017. Data was extracted from electronic patient records (Electronic patient record (EPR), clinical Information system (Metavision) and electronic drug charts (EPMA).

The inclusion criterion for the study was the use of terlipressin during the period of stay in PICU for pediatric patients admitted with liver disease. These records have been expressed as ‘patient administration records’ for this study. If terlipressin usage was interspersed by more than 15 days, it was taken as two separate entries, even if it was used in the same patient during same admission.

Patients were classified as having hepatorenal syndrome, if they satisfied the acute kidney injury (HRS-AKI) criteria as per the International Ascites Club definition 2012 [15, 16]. Nine patient administration records (of 7 patients), satisfied the diagnosis of HRS-AKI. All the remaining 9 patients had impairment of renal parameters in patients with liver disease, though they did not meet the definition of HRS-AKI, due to concomitant shock and requirement of inotropes After starting nor-adrenaline, when the renal parameters continued to deteriorate, terlipressin was started in these patients with non-HRS AKI.

The indications of terlipressin in non-HRS AKI included refractory vasodilatory shock (refractory to norepinephrine) and massive upper gastrointestinal bleed. Terlipressin was then administered either as an infusion or bolus, based on PICU consultant’s decision. Terlipressin was continued for 7–10 days based on the response (improvement of serum creatinine, increase of urine output) and was discontinued in case of any adverse effects or no improvement in serum creatinine/urine output after 4 days of starting terlipressin. We doubled the dose after 48 h if there was no response.

### Statistical analysis

Categorical data was presented as counts and percentage, and analyzed with help of chi square test. Skewed distribution was described as median and interquartile ranges. For parametric tests, t test (paired or unpaired) and for non-parametric tests, Wilcoxon signed rank test and Mann Whitney U test were used as appropriate.

## Results

### Baseline parameters

We extracted data for 21 terlipressin administration records, used in 16 patients with liver disease, who were administered terlipressin between 2010 and 2017. These patients had a median age of 10 years (6.1–14.4 years). All patients were treated in PICU (Table 1). Most patients had ascites (66.6%) and varices (76.19%). Seven patients were suggestive of hepatorenal syndrome-acute kidney injury (HRS-AKI) as diagnosed by International Ascites Club definition 2012 [15, 16].

**Table 1:** Baseline Parameters in patients prior to terlipressin initiation

Parameter	Median (IQR)
Age (in years)	10 (6.1–14.4)
Weight (in kg)	26 (17.5–45)
Sex (male children)	52.38%
Bleeding varices	16/21
Ascites	14/21
Concomitant noradrenaline usage	14/21(mean dose: 0.25 μg/kg/min)
Catecholamine index [17, 18]	10 (0.27–25)
Vasopressor dependency index [17, 18]	0.399 (0.089–0.87)
LIU score [19]	160 (115–141)
PIM score [20, 21]	4.4 (3.6–12.4)
PELOD score [22]	21 (12–31)
Hepatorenal syndrome-acute kidney injury	7/21
Baseline MAP (mmHg)	60 (52–68)
Baseline Urine output (ml/kg/hr)	0.055 (0–0.57)
Baseline Creatinine (μmol/L)	67 (36.5–97)
Baseline sodium (mmol/L)	138 (135–140)
Baseline lactate	2.24 (1.29–4.5)

*IQR* Interquartile range, *LIU* Liver injury unit scoring, *PIM* Pediatric index of mortality, *PELOD* Pediatric logistic organ dysfunction score, *MAP* Mean arterial pressure

Terlipressin was first given as a bolus in all cases, followed by either a continuous infusion (8 instances) or continued as boluses (13 instances). All patients had chronic liver disease. Of the 16 patients, 10 had acute on chronic liver failure (ACLF) with multiple organ failures and 6 patients had decompensated liver disease with ascites, bleeding varices and portal hypertension. Etiologies for hepatic impairment included genetic causes (as Alagille syndrome, hyperoxaluria), metabolic causes and biliary atresia (Table 2).

**Table 2:** Etiological Association of Terlipressin usage ^a^

Etiology	Total (*n* = 21)	Total who had HRS^c^
Decompensated liver disease	10	7/10
Genetic cause of liver disease	7	2/7
Previous transplant^b^	7	2/7^d^
Sepsis	7	1/7
Esophageal varices (with or without bleeding)	8	2/8

^a^Patient often had more than one condition at same time; ^b^ 7 case records had liver transplant terlipressin administration (6 before and 1 after)

^c^denominator is total with that particular etiology ^d^ one patient had liver transplant, and another intestinal transplant

#### Impact of terlipressin on renal parameters

**Fig. 1:**
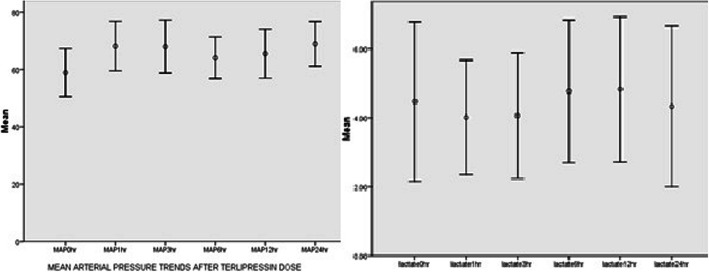
Mean arterial pressure and lactate trends over 1st 24 h after terlipressin administration

**Fig. 2:**
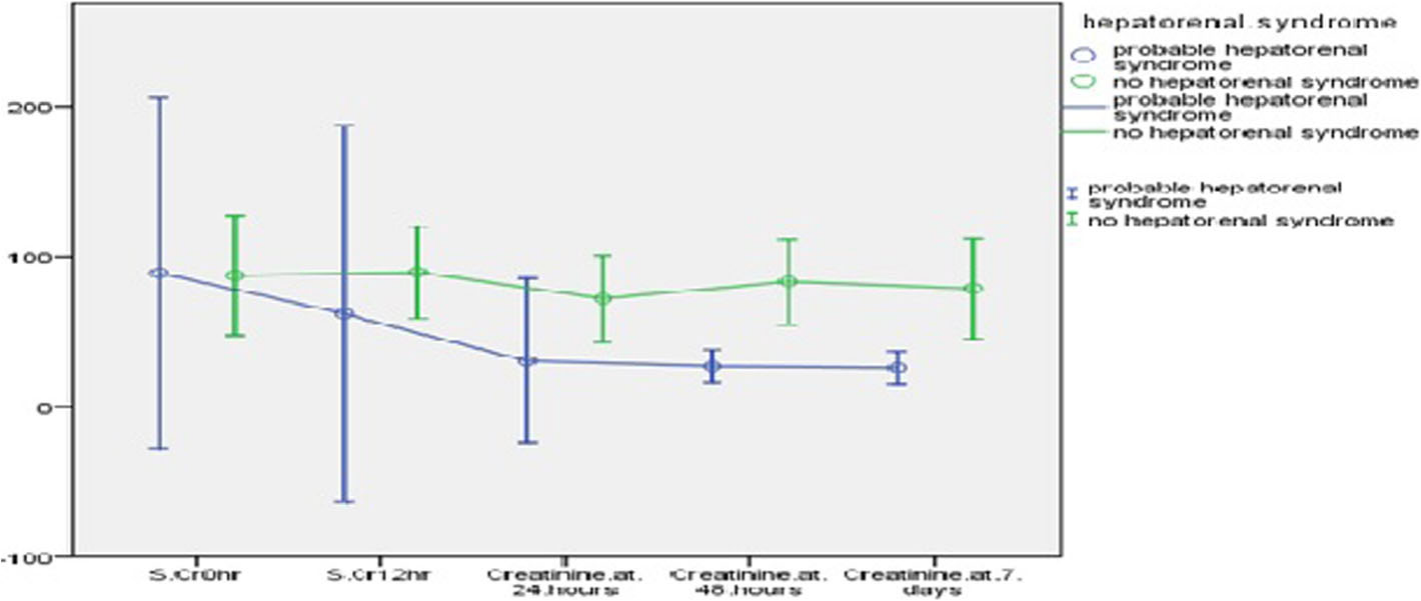
Comparison of creatinine response between HRS-AKI and non-HRS AKI

All patients included in the analysis satisfied the AKI definition of increased serum creatinine above the baseline. The impact on lactate and mean arterial pressure (MAP) are described in Fig. 1 and Table 3. A gradual improvement in serum creatinine was observed in the study period up to 7 days after starting terlipressin (Fig. 2). The median urine output prior to onset of treatment as 0.75 ml/kg/hr. (0–7.8)(median//IQR), which increased to 1.3 ml/kg/hr. (0–6.9).

**Table 3:** Profile of Terlipressin usage for HRS-AKI patients^a^

Parameter	HRS ***n*** = 7 pts (9 patient records)	Non HRS ***n*** = 9 pts (15 patient records)	***p*** value
Renal angina index	10 (1–10)	5 (5–8)	0.06
Creatinine 0 h^b^	84 (48–116)	67 (38–78)	0.43
Creatinine 12 h^b^	36 (30–120)	70 (48–108)	0.53
Creatinine 24 h^b^	36 (17–41)	58.5 (33–89.5)	0.39
Creatinine 48 h^b^	29 (22–30)	71 (37–96.5)	0.32
Creatinine 7 days^b^	28 (23–29)	65.5 (25–85.5)	0.32
Urine output 0 hb^c^	0.6 (0–1.1)	0.1 (0–4)	0.43
Urine output 6 hb^c^	2.6 (0.2–5.0)	0.2 (0–1.9)	0.1
Urine output 12 hb^c^	1.5 (0–3.0)	0 (0–1)	0.392
Urine output 24 hb^c^	0.9 (0–1.8)	0.3 (0–1.7)	0.42
Urine output 48 h^cb^	3 (0–3)	2 (0–2)	0.029
Urine output 7 daysb^c^	2 (0–2)	3 (0–4)	0.306
Sodium 0 h^dc^	141 (136–152)	138 (135–139)	0.202
Sodium 12 h^dc^	150.5 (143–158)	136.4 (134.6–139)	0.191
Sodium 24 h^dc^	143 (137.5–155)	137 (135–138)	0.259
Sodium 48 h^dc^	138 (135–139)	140 (136–144)	0.354
Sodium 7 daysc^d^	138 (137–138)	138 (137–142)	0.696
Mortality	40%	50%	0.55

^a^patient administration records; ^b^expressed as (median)(IQR); *Cr* Creatinine expressed in μmol/L;^cb^ in ml/kg/hr.; ^dc^ in meq/litre

#### Adverse effect profile

There were 3 adverse events observed following administration. These included 2 incidents of digital ischemia (blanching or blueness of extremities) and one incident of feeding intolerance. In one instance, the drug had to be discontinued because of adverse effect. No significant effect on lactate levels was observed (Baseline - 3.3(0.7–11.23), it remained constant, 3.48(1.1–10.6) at 1 h(*p* = 0.45) and 3.4(0.51–11.6) at 6 h(*p* = 0.688). There was no significant effect on serum sodium as well [(0 h: 138 meq/litre (135.1–140.5); 6 h: 139(135–143)(*p* = 0.526);12 h:136.9(134.8–141.5)(*p* = 0.567);24 h 137.5(136–140)(p:0.609)] This is shown in Table 3.

### Role in hepatorenal syndrome (HRS-AKI)

There was an improvement in urine output and serum creatinine after terlipressin administration, but the difference between the groups was not significant (Table 3 and Fig. 2). Both groups reported adverse effects (2 episodes of digital ischemia in non HRS group, and I episode of feeding intolerance in HRS group). Of the 7 patients with HRS, 5 responded with a decrease in serum creatinine at 48 h (response rate of 71.4%) whereas of the 9 patients with shock and on inotropes, only 4 responded with decrease in serum creatinine and increase of urine output (response rate of 33.3%).

#### Terlipressin dose

In our case series, the drug was initially given as a bolus dose in all cases, followed by either bolus or infusion. Doses are hence expressed as mcg/kg/hour, to account for this difference. Most administrations were as bolus doses of 1 mg (0.5–2 mg) at the start of drug use.

The average duration of bolus use was 40 h (0–60), with median of 5 boluses (range: 1–44) received. The median dose (IQR) used was 5.2(3.8–6.7 mcg/kg/hr). In HRS-AKI we used terlipressin at the dose of 5.2 (4.5–6.4) (IQR) mcg/kg/hr.

### Outcome

Of the 16 patients, 7 patients died, mortality of 47.5%. Of these, 2 patients had HRS-AKI, giving a mortality of 28.5% to this group. There was no statistical difference in urine output, creatinine and mean arterial pressure, between the survivors and non-survivors. Multiple causes contributed to this mortality - gastrointestinal bleeding (in 4 patients), multiple organ dysfunction syndrome (MODS) (in 6 patients) and sepsis (in 2 patients).

## Discussion

Terlipressin is a powerful V1(vasopressin) receptor agonist [17]. At our center, we use terlipressin for various indications, which range from hepatorenal syndrome, septic shock to variceal bleeding.

### Renal parameters

Splanchnic arterial vasodilation is considered important in the pathogenesis of HRS-AKI. Use of vasoconstrictive drugs results in splanchnic arteriolar vasoconstriction with decreased portal pressure, thereby resulting in redistribution of blood flow, thereby improving renal blood flow [18–20]. Vasopressin analogues (ornipressin, terlipressin), octreotide and noradrenaline have been used for the same effect.

Adult studies have demonstrated improvement in renal parameters. When terlipressin is used in hepatorenal syndrome, reversal of HRS defined as decrease in serum creatinine by 50% baseline to less than 1.5 mg/dl is demonstrated in 45–55% cases [10, 11, 20]. In our study population as well, we observed a drop in serum creatinine and increase in urine output in the HRS-AKI population as compared to non HRS-AKI patients. In our experience, terlipressin use in this subgroup, was safe, with minor side effects. Adult literature also confirms the safety profile of terlipressin when used in HRS-AKI patients [10, 11, 21–23]. .Looking at the response rate in HRS AKI versus those in shock, it might give us a clue to use terlipressin only in patients where there the patient satisfies the clinical definition of HRS-AKI.

#### Dose of terlipressin

There is a paucity of literature on pediatric terlipressin usage. We could only find a single case series, where the average unique dose of 15 to 20mcg/kg 4-hourly was used. All of the patients in this case series received terlipressin as a continuous infusion at 30 mcg/kg/day [14]. In our case series, we predominantly used bolus doses while using infusion in some. (Median dosage 5.2 (3.8–6.7 mcg/kg/hour).

### Side effect profile of terlipressin /vasopressin analogues

Most of the side-effects of terlipressin are due to its severe vasoconstrictive properties. In our study, we found minimal evidence of gastrointestinal side effects (including feeding intolerance) and vasoconstrictive side effects including cyanosis of extremities.

There have been many adult case reports of severe side effects after terlipressin usage, including severe skin necrosis (after extravasation of low dose vasopressin) [1, 24] gastrointestinal ischemia [1, 25], coronary ischemia and increased risk of thrombosis [26]. The dose of terlipressin depends upon the indication for the which the drug is being used and individual patient profile. Certain side effects may necessitate discontinuation of the drug.

Though previous studies have reported metabolic side effects such as hyponatremia and features of microvascular ischemia with lactic-acidosis, this was not observed in our study [1, 25].

### Limitations

This is a single center retrospective study of patients, hence, it carries the limitations of risk of bias and lack of complete records due to retrospective nature of data collection. In addition, the vital parameters, were recorded with a focus on first 24 h of starting the drug. The biggest challenge is retrospectively classifying patients with HRS AKI versus those with shock, though proven to be non-pre-renal. HRS in paediatrics is not very well defined. It is very difficult to prove the efficacy in terms of bridging to transplant. Does improvement in biochemical parameters equate to improved survival, cannot be answered by our data. It is also limited by the small sample size and use of terlipressin in those patients who were on renal replacement therapy. A prospective, adequately powered, placebo controlled, multicenter trial may be beneficial in validating these results.

## Conclusions

To conclude, our experience of using terlipressin in critically ill children with liver disease shows that terlipressin is safe and might be useful in decreasing serum creatinine especially in children with HRS-AKI. Whether reduction in serum creatinine and increase in urine output translates to improved clinical outcomes both in terms of reversal of HRS and improved survival (with or without transplant) remains to be seen. An important conundrum which needs to be answered is whether prolonged terlipressin usage can be used to as a bridge to liver transplantation in this cohort of sick liver patients. It is important to be aware of the side-effect profile and expectantly manage them. Future research is needed to validate the findings of this study.

## Data Availability

The dataset used and/or analysed during the current study are available from the corresponding author on reasonable request.

## Abbreviations

HRS-AKI: Hepatorenal Syndrome Acute Kidney Injury
EVH: Esophageal Variceal Hemorrhage
PICU: Paediatric Intensive Care Unit
IQR: Interquartile Range
Mcg: Micrograms
Kg: Kilograms
Hr: Hours
Cr: Creatinine
UO: Urine Output
Min: Minutes
Ml: Millilitres
EPR: Electronic Patient Record
EPMA: Electronic Drug Charts
